# Genetic mutations in the treatment of anaplastic thyroid cancer: a systematic review

**DOI:** 10.1186/1471-2482-13-S2-S44

**Published:** 2013-10-08

**Authors:** Anna Guerra, Vincenzo Di Crescenzo, Alfredo Garzi, Mariapia Cinelli, Chiara Carlomagno, Massimo Tonacchera, Pio Zeppa, Mario Vitale

**Affiliations:** 1 Medicine and Surgery, University of Salerno, Salerno, Italy; 2 Public Health, University of Naples "Federico II", Naples, Italy; 3 Clinical Medicine and Surgery, University of Naples "Federico II", Naples, Italy; 4 Endocrinology, Research Center of Excellence AmbiSEN, University of Pisa, Pisa, Italy

## Abstract

**Background:**

Anaplastic thyroid cancer (ATC) is a rare, lethal disease associated with a median survival of 6 months despite the best multidisciplinary care. Surgical resection is not curative in ATC patients, being often a palliative procedure. Multidisciplinary care may include surgery, loco-regional radiotherapy, and systemic therapy. Besides conventional chemotherapy, multi kinase-targeted inhibitors are emerging as novel therapeutic tools. The numerous molecular alteration detected in ATC are targets for these inhibitors. The aim of this review is to determine the prevalence of the major genetic alterations occurring in ATC and place the results in the context of the emerging kinase-targeted therapies.

**Methods:**

The study is based on published PubMed studies addressing the prevalence of BRAF, RAS, PTEN, PI3KCA and TP53 mutations and RET rearrangements in ATC.

**Results:**

21 articles dealing with 652 genetic analyses of the selected genes were used. The overall prevalence determined were the following: RET/PTC, 4%; BRAF, 23%; RAS, 60%; PTEN, 16%; PI3KCA, 24%; TP53, 48%. Genetic alterations are sometimes overlapping.

**Conclusions:**

Mutations of BRAF, PTEN and PI3KCA genes are common in ATC, with RAS and TP53 being the most frequent. Given ATC genetic complexity, effective therapies may benefit from individualized therapeutic regimens in a multidisciplinary approach.

## Introduction

Thyroid cancer is the most prevalent endocrine malignancy accounting for 1% of cancers worldwide. More than 95% of thyroid cancer are well differentiated tumors that respond to surgery followed by radioactive iodine (RAI) therapy and thyroid hormone suppression. Although disease recurrence occurs in approximately 30% of cases, nowadays thyroid cancers have a very favorable outcome. The clinical appearance of thyroid cancer is that of a nodules, some time representing a challenging diagnostic dilemma with thyroid or unusual extrathyroidal masses [1,2]. The use of effective diagnostic tools such as ultrasound (US) and fine-needle cytology (FNC) [3-5] has increased the detection of small and well differentiated tumors in their early stages. Moreover, the application of molecular techniques to FNC has dramatically increased its sensitivity [3,4,6-9]. An effective FNC diagnosis avoids useless diagnostic surgery or provides indications for the proper surgical treatment, when needed [10,11]. Poorly differentiated subtypes, including anaplastic thyroid cancer (ATC), are resistant to RAI and conventional chemotherapy. ATC accounts for about 1% of thyroid cancer and is typical of old age. When feasible, surgery must aim at a radical intent; however, surgical resection is not curative in ATC patients, being often a palliative procedure [10,11]. Therefore, an early and accurate diagnosis is mandatory in case of ATC which does not require surgical treatment, and even more in elderly patients, for whom surgery is generally more burdensome, complex and expensive than younger patients [10,11]. Standard chemotherapies have systemic toxicities and limited efficacy in the case of ATC as well as of other more common solid tumors [12-14]. Alternative strategies such as immunotherapy are under investigation, but still far from clinical practice [15]. At present, genetic-based targeted therapy is the most promising curative strategy. Hallmarks of all cancers are self-sufficiency in growth signals and evasion of programmed cell death. Tyrosine kinase receptors/RAS/RAF/MAPK and RAS/PI3K/Akt/mTOR are the major signaling pathways involved in cell proliferation, protein synthesis and cell survival. Thyroid cancer is characterized by several genetic alterations along these two pathways, including rearrangements of the *RET *(rearranged during transfection; *RET/PTC*) tyrosine receptor kinase, activating point mutations in the *BRAF *serine/threonine kinase, in the *RAS *proto-oncogenes, in the catalytic subunit of the phosphatidyl-inositol 3-Kinase (*PI3KCA*), or inactivating mutations in the tumor suppressors phosphatase and tensin homolog (*PTEN*) and *TP53 *(Table 1). ATC is the product of the accumulation of genetic alterations due to genetic instability and external factors such as food or environmental factors, including ionizing radiations and oxidative stress. Oxidative stress has been implicated in the mechanism of cancer, diabetes, cardiovascular and other diseases [16,17]. Oxidant molecules are generated by stress agents such chemicals, drugs, pollutants, and high-caloric diets [18]. Conversely, there is no hint of a remodeling of the Ca^2+ ^toolkit, that has been observed in other malignancies, including renal cellular carcinoma [19-21], and prostate cancer [22], and has been put forward as alternative target for selective molecular therapies [14]. The last decade has seen advances in the understanding of the molecular basis of thyroid cancer, leading to the application of new pharmacological treatments with inhibitors of kinases [23-25]. These drugs are multi-target agents with inhibitory activity of receptors involved in the angiogenesis or inhibitors of kinases involved in thyroid cancer development. The BRAF inhibitor vemurafenib (PLX4032) improves survival among patients with metastatic melanoma, and suppresses growth of BRAF-mutated human ATC in a mouse model [26]. The beneficial effect of BRAF inhibition in ATC with activating BRAF mutations has been recently reported [27]. Other pharmacological compounds inhibit RET and RET/PTC (sorafenib, sunitinib, vandetanib) or the mammalian target of rapamycin (mTOR), a component of the PI3K/Akt signaling pathway (everolimus). Hence, the knowledge of the tumor mutation status is needed for optimizing and tailoring the treatment with kinase inhibitors. The intent of this systematic review is to determine the prevalence of the major genetic alterations occurring in ATC.

**Table 1:** Gene mutations in ATC

Gene	Mutation	Signaling involvement
*RET*	Recombination	MAPK activation
*BRAF*	Single point mutation	MAPK activation
*H-, N-, K-RAS*	Single point mutation	MAPK, PI3K/Akt/mTOR activation
*PTEN*	Single point mutation/deletion	PI3K/Akt/mTOR inactivation
*PI3KCA*	Single point mutation	PI3K/Akt/mTO activation
*TP53*	Single point mutation	P53 pathway inactivation

## Materials and methods

A meta-analysis was performed by searching the MEDLINE database (National Library of Medicine, Bethesda, MD) using the terms "BRAF", "RAS", "PTEN", "PI3KCA", "TP53", "RET/PTC" or ''BRAF,'' associated with the terms ''anaplastic thyroid cancer" or "undifferentiated thyroid cancer''. Studies were included only when the sample was ≥ 4. Studies were selected on the basis of the detection of molecular alterations by genetic analysis. Studies based only on molecular detection by immunohistochemistry were excluded. Only data about different genes were included from studies by the same authors. Studies on poorly differentiated thyroid cancers and well differentiated thyroid cancers were also excluded.

## Results

The literature search strategy retrieved 104 articles from PubMeD. Twenty-one studies met the inclusion criteria and were considered for further analysis. These studies were published between 1993 and 2010, and included 652 cases of ATC. All studies were retrospective, using stored formalin-fixed paraffin-embedded samples or frozen surgical specimens. The method used for determining the presence of single point mutations was direct sequencing of DNA after polymerase-chain reaction (PCR) amplification, PCR and fluorescence melting curve analysis and DNA-mutant allele-specific amplification (DNA-MASA). The methods used to determine *RET *rearrangements were PCR alone followed by direct sequencing or PCR followed by internal probe binding (Southern blot on PCR products). *BRAF^V600E ^*was the only *BRAF *mutation considered by the 7 studies analyzed. The mutation ranged 0%-50% in 21 out of 89 tumors (Table 2). The mean prevalence was 23%. Mutations in the three *RAS *isoforms ranged 8%-60% in 33 out of 162 ATCs (mean 60%). Not all the three major *RET *rearrangements were considered in all studies. Tumors were tested for the presence of *RET/PTC-1 *and -3 in two studies and *RET/PTC-1, -2*, and *-3 *in one study. Rearrangements were rare, being detected in 4% of ATCs, in the range 0%-6% in 3 out of 81 tumors. Inactivating mutations of *PTEN *were detected in 16% of 107 ATCs, while activating mutations of PI3KCA in 23% of 70 ATCs in the range 12%-58% (Table 3). Inactivating mutations of TP53 were identified in 48% of 25 tumors, in the range 10%-86%.

**Table 2:** Prevalence of mutations in the MAPK pathway in ATC

Mutation	Positive/ total cases	Prevalence (%)	Reference
*BRAF^V600E^*	0/7	0	[51]
	2/6	33	[52]
	3/29	10	[53]
	2/10	20	[54]
	8/16	50	[55]
	0/4	0	[56]
	6/17	35	[57]
Overall *BRAF^V600E^*	21/89	23	

*RAS*	4/50	8	[44]
	2/18	11	[58]
	1/5	20	[59]
	4/18	23	[43]
	15/29	55	[60]
	4/50	8	[61]
	3/5	60	[62]
Overall *RAS *mutations	33/162	20	

*RET/PTC*	0/14	0	[63]
	3/51	6	[44]
	0/17	0	[64]
Overall *RET/PTC*	3/81	4	

**Table 3:** Prevalence of mutations not in the MAPK pathway in ATC

Mutation	Positive/ total cases	Prevalence (%)	Reference
*PTEN*	8/48	17	[44]
	8/50	16	[61]
	1/9	10	[65]
Overall *PTEN*	17/107	16	

*PI3KCA*	6/50	12	[44]
	4/18	22	[58]
	29/50	58	[61]
	16/70	23	[66]
Overall *PI3KCA*	45/188	24	

*TP53*	1/11	10	[67]
	5/7	71	[19]
	6/7	86	[68]
Overall *TP53*	12/25	48	

## Discussion

The prognosis of differentiated thyroidal tumors is generally favorable mainly because there are different and effective tools in the early diagnosis and treatment of these tumors [28]. In fact, the use of US and FNC in the diagnosis of thyroid nodules usually leads to an early and accurate diagnosis of small and differentiated tumors, as well as less frequent thyroidal neoplasms [3,5,6]. In particular FNC, coupled with immunocytochemistry (ICC), flow cytometry (FC) and molecular techniques [3-6,29-31] has dramatically enhanced the sensitivity and the accuracy of preoperative diagnosis of thyroidal nodules [3,5,29]. The bad prognosis of advanced thyroid carcinoma, prompted researchers to evaluate the efficacy of new pharmaceutical compounds with enzymatic inhibitory properties (Table 4). The prevalence of *RET/PTC *rearrangements in ATC was much lower than in papillary thyroid cancer reported in most of the studies (4% vs. 36%) [25,32]. Noteworthy, benign thyroid nodules exhibiting *RET/PTC *rearrangements do not evolve in cancer [33,34]. This data suggest that this oncogene has a minor role in the progression from well-differentiated to undifferentiated thyroid cancer. It also indicate that tyrosine kinase inhibitors such as sorafenib, sunitinib, and vandetanib have little chance to function through the inhibition of this oncogene in ATC. The encouraging results obtained by these drugs in non RAI-responsive differentiated thyroid carcinomas in some clinical trials where the *RET *rearrangement was not evaluated, were more likely due to the effects on neo-angiogenesis [35]. The high prevalence of *BRAF^V600E ^*mutation in ATC supports the hypothesis that many ATCs actually represent a progressive malignant degeneration of *BRAF*-mutated, well-differentiated thyroid carcinomas [36]. This gene is a pivotal component of the MAPK pathway and reduces the activity of p21^kip1 ^in thyroid tumors, stimulating the cell cycle machinery [37]. Vemurafenib (PLX4032), a BRAF selective kinase inhibitor and sorafenib, a multi-target inhibitor, find application in selected BRAF-mutation positive melanomas [38]. Although clinical studies of BRAF inhibitors in advanced non RAI-responsive differentiated thyroid carcinomas have shown encouraging results with frequent early responses, in a relevant fraction of patients this effect was of limited duration, with frequent relapse or no response. In addition, intratumoral heterogeneity with respect to *BRAF *mutation makes the evaluation of these clinical trials even more complex [39,40]. Poor results were obtained with sorafenib in ATC, although positive results reported with vemurafenib in one ATC with *BRAF^V600E ^*mutation are worthy to be mentioned [27,35]. A relevant obstacle to the efficacy of treatments based on the inhibition of BRAF^V600E ^is the presence of activating mutations of *RAS*. This proto-oncogene is a small GTP binding protein located upstream RAF in the MAPK cascade. Activating mutations of this protein reactivate the MAPK pathway, making *BRAF^V600E ^*inhibition inefficient [41]. The high prevalence of *RAS *activating mutations in ATC (60%) makes the inhibition of the MAPK pathway by kinase inhibitors a strategy whose success is unlikely. Moreover, papillary thyroid carcinoma and ATC exhibit concomitant *BRAF^V600E ^*and *RAS *mutations, although a rare occurrence [42-44]. In light of these considerations, the pharmacological inhibition of the MAPK pathway looks less promising than the inhibition of the PI3K/Akt/mTOR pathway. This pathway is constitutively activated by inactivating mutations of PTEN and by activating mutations of PI3KCA. Both mutations are frequent in ATC (10% and 24% respectively). Ongoing studies in cells, both in culture and *in vivo*, are investigating the anticancer effect of the novel allosteric Akt inhibitor, MK2206, in combination with several anticancer agents [45]. This agent selectively inhibits thyroid cancer cells harboring mutations that can activate the PI3K/Akt pathway [46,47]. An appealing feature of Akt/mTOR inhibitors is the possibility of treating advanced thyroid cancer also when resistance to single targeted therapy is conferred by multiple genetic alterations. Most of the kinase inhibitors currently under investigation are multitargeted inhibitors, with a beneficial double effect impairing the viability of tumor cells and tumor vascularization [13,14,20,48]. The TP53 tumor suppressor gene increases the cyclin kinase inhibitor p21^kip1^, promoting cell cycle arrest at G1/S. Its inactivation by a mutation impairs the correct modulation of cell proliferation and apoptosis. This gene is mutated in 48% of ATC. The loss of the TP53 mediated control of the apoptotic machinery is probably the most difficult obstacle to overcome for a pharmacological agent to be active in ATC. Beneficial effects in ATC cell lines have been observed with an adenovirus TP53-regulated Cre/loxP system and with a E1B gene-defective adenovirus (ONYX-015) in TP53 mutant cells [49,50].

**Table 4:** Major pharmaceutical compounds in clinical development for the treatment of thyroid cancer

Pharmaceutical compound	VEGFRs	RET/PTC	BRAF	PDFGR	mTORC1
Axitinib	+		-	+	-
Cabozantinib	+/-		-	-	-
Lenvatinib	+		-	+	-
Motesanib	+		-	+	-
Pazopanib	+		-	+	-
Sorafenib	+	+	+	+	-
Sunitinib	+	+	-	+	-
Vandetanib	+	+	-	-	-
Vemurafenib	-	-	+	-	-
Everolimus	-	-	-	-	+

## Conclusions

ATC is characterized by genomic instability that leads to mutations in *RET, BRAF, RAS, PTEN, PIK3CA *and *TP53 *genes. The survival of ATC patients has changed little in the past 50 years, despite the introduction of new therapeutic tools. Given the complexity of the genomic alterations of ATC, therapy results may benefit from individualized therapeutic regimen that maximally inhibits major pathways. In the future, these therapies may be successful with a multidisciplinary approach.

## Competing interests

The authors declare that they have no competing interests.

## Authors' contributions

MV: conception and design, interpretation of data, AG, PZ, VDS, AG, MPC: acquisition of data, drafting the manuscript, PZ, MV: critical revision, given final approval of the version to be published.

## Authors' information

AG = Resident in Clinical Pathology at University of Salerno

VDC = Aggregate Professor of Thoracic Surgery at University of Salerno

AG = Aggregate Professor of Pediatric Surgery at University of Salerno

MC = Aggregate Professor of Anatomy, University of Naples "Federico II"

CC = Aggregate Professor of Oncology, University of Naples "Federico II"

MT= Associate Professor of Endocrinology at University of Pisa

PZ= Associate Professor of Pathology at University of Salerno

MV= Associate Professor of Endocrinology at University of Salerno

## Declarations

Funding of this project has come from personal funds.

This article has been published as part of *BMC Surgery *Volume 13 Supplement 2, 2013: Proceedings from the 26th National Congress of the Italian Society of Geriatric Surgery. The full contents of the supplement are available online at http://www.biomedcentral.com/bmcsurg/supplements/13/S2
